# PPG Signals-Based Blood-Pressure Estimation Using Grid Search in Hyperparameter Optimization of CNN–LSTM

**DOI:** 10.3390/diagnostics13152566

**Published:** 2023-08-01

**Authors:** Nurul Qashri Mahardika T, Yunendah Nur Fuadah, Da Un Jeong, Ki Moo Lim

**Affiliations:** 1 Computational Medicine Lab, Department of IT Convergence Engineering, Kumoh National Institute of Technology, Gumi 39177, Gyeongbuk, Republic of Korea; nurulqika@kumoh.ac.kr (N.Q.M.T.); yunendah@kumoh.ac.kr (Y.N.F.); dawny6960@kumoh.ac.kr (D.U.J.); 2 School of Electrical Engineering, Telkom University, Bandung 40257, Indonesia; 3 Computational Medicine Lab, Department of Medical IT Convergence Engineering, Kumoh National Institute of Technology, Gumi 39177, Gyeongbuk, Republic of Korea; 4 Meta Heart Co., Ltd., Gumi 39177, Gyeongbuk, Republic of Korea

**Keywords:** photoplethysmography (PPG), blood pressure, grid search, convolutional neural network, long short-term memory

## Abstract

Researchers commonly use continuous noninvasive blood-pressure measurement (cNIBP) based on photoplethysmography (PPG) signals to monitor blood pressure conveniently. However, the performance of the system still needs to be improved. Accuracy and precision in blood-pressure measurements are critical factors in diagnosing and managing patients’ health conditions. Therefore, we propose a convolutional long short-term memory neural network (CNN–LSTM) with grid search ability, which provides a robust blood-pressure estimation system by extracting meaningful information from PPG signals and reducing the complexity of hyperparameter optimization in the proposed model. The multiparameter intelligent monitoring for intensive care III (MIMIC III) dataset obtained PPG and arterial-blood-pressure (ABP) signals. We obtained 75,226 signal segments, with 60,180 signals allocated for training data, 12,030 signals allocated for the validation set, and 15,045 signals allocated for the test data. During training, we applied five-fold cross-validation with a grid-search method to select the best model and determine the optimal hyperparameter settings. The optimized configuration of the CNN–LSTM layers consisted of five convolutional layers, one long short-term memory (LSTM) layer, and two fully connected layers for blood-pressure estimation. This study successfully achieved good accuracy in assessing both systolic blood pressure (SBP) and diastolic blood pressure (DBP) by calculating the standard deviation (SD) and the mean absolute error (MAE), resulting in values of 7.89 ± 3.79 and 5.34 ± 2.89 mmHg, respectively. The optimal configuration of the CNN–LSTM provided satisfactory performance according to the standards set by the British Hypertension Society (BHS), the Association for the Advancement of Medical Instrumentation (AAMI), and the Institute of Electrical and Electronics Engineers (IEEE) for blood-pressure monitoring devices.

## 1. Introduction

Two methods are available for continuously measuring blood pressure: invasive and non-invasive. The invasive method involves healthcare professionals inserting a cannula needle into the artery. This method is used, for example, for patients in intensive care units (ICUs) with acute functional impairment [1]. However, this method can be inconvenience and cause side effects such as infection, local bleeding, arterial obstruction, distal limb ischemia, and vascular injury. Non-invasive techniques such as sphygmomanometers (cuff-based monitoring) involve inflating a cuff and auscultating blood pulsations, which may cause arterial compression and which requires precise procedures to obtain continuous and accurate measurements [2].

Recently, researchers have been using photoplethysmography (PPG) signals to estimate blood pressure. PPG utilizes the optical signal from light reflected from body tissues (blood vessels) to measure volumetric changes during the cardiac cycle [3,4,5,6]. PPG sensors emit light onto body tissues and detect the reflected intensity. As blood circulates through the vessels, the changes in blood volume cause variations in the intensity of reflected light, resulting in the PPG waveform. Therefore, PPG signals provide valuable information about pulse rates and characteristics of blood-pressure waveforms related to the cardiac cycle. 

Several studies have focused on analyzing blood pressure using important features extracted from PPG signals. These features include morphological characteristics, frequency components, and the relationship between peaks and troughs in PPG signals [7,8,9,10,11,12]. Therefore, machine-learning or deep learning techniques have been employed to utilize the information from PPG signals to generate accurate and reliable blood-pressure predictions.

Zhang et al. and Hasanzadeh et al. proposed conventional machine-learning models for blood-pressure estimation using PPG features. Zhang et al. focused on unclear dichotic pulse waveforms and used parameters such as systolic upstroke time (ST), diastolic time (DT), and pulse width as features. The mean absolute errors (MAEs) for predicting systolic blood pressure (SBP) and diastolic blood pressure (DBP) were 11.6 and 7.6, respectively—however, the proposed model had to meet the Institute of Electrical and Electronics Engineers (the IEEE) standard [7]. Hasanzadeh et al. utilized a morphological analysis of the PPG signal. They achieved a performance that met the standards of the Association for the Advancement of Medical Instrumentation (the AAMI) for SBP and DBP prediction [8]. However, their study had a small sample size and did not meet the minimum standards set by the IEEE, and no comparison with existing models was conducted.

Furthermore, Samimi et al., in their study, utilized interval-beat PPG signals from the MIMIC II dataset to analyze the differences between the peak and trough points of the PPG signal. The average value of these differences was employed to divide the signal into distinct characteristic segments. Their study aimed to evaluate the accuracy of the mean peak-to-peak (mPTP) features in estimating blood pressure (BP). Among several proposed scenarios, their study reported the MAE + SD performance values for DBP and SBP as 4.65 mmHg ± 12.40 mmHg and 5.59 mmHg ± 7.35 mmHg, respectively. However, their study had limitations, including unsatisfactory performance in estimating blood pressure and failure to meet the minimum standards set by the IEEE [13]. 

Several studies also adopted featureless and deep learning techniques in estimating systolic blood pressure (SBP) and diastolic blood pressure (DBP) based on PPG signals. This approach avoids using complex features and leverages the ability of neural networks to learn hidden patterns in the signals. A study by Slapnicar et al. applied the residual network (ResNet) approach using PPG signals, first-order PPG derivatives, and second-order PPG derivatives from the MIMIC III dataset. Each signal was segmented into 5 s intervals and fed into the proposed model. The study achieved MAE performances of 9.43 mmHg for SBP estimation and 6.80 mmHg for DBP estimation [10]. However, the use of residual networks is more sensitive to the choice of hyperparameters, and it training can be more challenging because of the complex architecture of residual networks. In addition, multiple hyperparameters need to be determined, such as the number of residual blocks, network depth, and dropout rate [14,15]. Incorrect choices of these hyperparameters can lead to overfitting or underfitting the blood-pressure-estimation data.

Ibtehaz et al. focused on transforming PPG signals to arterial blood pressure (ABP) waveforms, while conserving the waveform characteristics, to determine SBP and DBP [11]. In their study, the MAEs for SBP and DBP were 3.45 mmHg and 5.73 mmHg, respectively. However, the proposed PPG2ABP pipeline, utilizing two one-dimensional (1D) CNN-based segmentation networks, may introduce computational complexity and additional resource requirements. Using deep learning models in the pipeline demands substantial computational power and longer processing times, potentially limiting its real-time applicability. 

Another approach, by Aguirre et al., adopted a method that utilized only 5 s PPG signals in a recurrent neural network (RNN) model, specifically the RNN encoder and decoder. Their study obtained MAEs of 6.57 mmHg for SBP prediction and 14.39 mmHg for DBP prediction [12]. However, neither SBP nor DBP met the IEEE standards. This discrepancy may be attributed to the intricate structures of the RNN encoder and decoder, which involve the incorporation of frequency in the temporal data processing. Consequently, this process entails long sequences and a high computational time, which affect the performance and stability of the model.

Li et al. applied an approach that utilized 700 data points from PPG signals in the multiparameter intelligent monitoring for intensive care II (MIMIC II) dataset and employed a combined CNN and bidirectional LSTM model (CNN–BiLSTM) for blood-pressure estimation. While this approach demonstrated potential in blood pressure estimation, using excessively long data points in deep learning models can significantly increase computational time, mainly when dealing with complex models such as CNN–BiLSTM [16].

Based on the relatively large mean absolute error (MAE) values, the previous studies’ performance in predicting systolic blood pressure (SBP) and diastolic blood pressure (DBP) has yet to reach the expected standards. Moreover, some previous research has employed complex techniques, such as deep learning with manually selected hyperparameter. These tuning processes involve determining the number of residual blocks, the network depth, and the dropout rates. They can lead to overfitting or underfitting issues in blood-pressure estimation data if not properly optimized. Consequently, the methods exhibit low performance while the computational time increases, leading to longer processing durations. Therefore, to address these issues, we proposed the CNN–LSTM model with a grid-search method for automatic hyperparameter tuning in specific parameter settings to reduce computational time.

The convolutional neural network (CNN) exhibits effective capabilities in feature extraction from data through convolutional operations to identify specific patterns. Another advantage of the CNN is its ability to perform parallel data processing, which increases efficiency in interpreting and analyzing dimension-reduced data [17]. On the other hand, LSTM plays a crucial role in capturing temporal dependencies and long-term patterns in sequential data. The combination of CNN and LSTM allows the CNN–LSTM model to efficiently and effectively handle sequential data, including complex blood-pressure estimation tasks. Therefore, the proposed approach possesses significant potential in achieving the main goal of this study by providing a highly accurate and reliable computational system for efficient blood-pressure estimation.

Configuring parameters is crucial in achieving optimal machine-learning performance. Hyperparameter tuning aims to find the combination of hyperparameter values that yield the most accurate prediction estimates among all the analyzed possibilities. However, manual parameter adjustment is time-consuming, particularly when the model involves many parameters. Furthermore, it is essential to consider the significance of proper parameter settings for achievable prediction performance. Therefore, we address these issues by employing the grid-search method, which automatically selects the best parameters for each machine-learning model. This approach enables us to optimize the model’s performance and reduce the time required for manual parameter adjustment.

## 2. Materials and Methods

**Figure 1:**
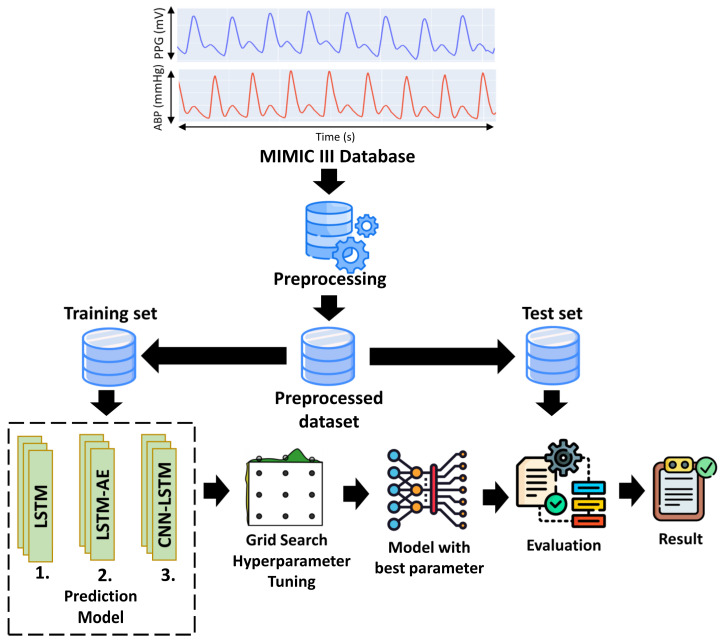
The proposed general block diagram system.

Figure 1 shows the proposed method for estimating SBP and DBP using one-dimensional (1D) PPG signals. After the preprocessing step, the PPG signals were split into training and testing sets. The proposed architecture (LSTM, LSTM–autoencoder (LSTM–AE), and CNN–LSTM) was trained using the training set as the input data, and grid search was used for hyperparameter tuning to determine the optimal parameters. Furthermore, the best model was tested using a test set to estimate SBP and DBP values.

### 2.1. Dataset 

Simultaneous PPG and ABP signals were acquired with a sampling frequency of 125 Hz and 8-bit precision from the multiparameter intelligent monitoring for intensive care III (MIMIC III) dataset, accessible on the PhysioNet website. The MIMIC III database comprises patients in the intensive care unit (ICU), collected by the Beth Israel Deaconess Medical Center (Boston, MA, USA) and the Massachusetts Institute of Technology (Cambridge, MA, USA) [18]. 

### 2.2. Preprocessing

**Figure 2:**
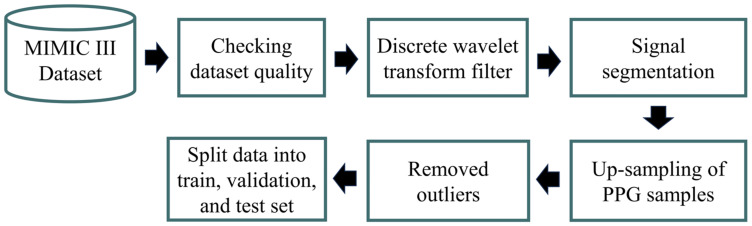
The proposed preprocessing method.

The preprocessing involved several steps, as shown in Figure 2. We utilized Jupyter Notebook with Python 3.9 and an Intel(R) Core(TM) i7-10700F CPU @2.80 GHz for preprocessing and prediction tasks. MIMIC III was extracted via WFDB, a Python library supported by Physionet. Furthermore, to address the presence of low-quality recordings containing flatlines and flat peaks arising from data acquisition issues, we automatically removed files containing incomplete ABP and PPG waveform shapes and flat peaks (referred to as ABP and PLETH). As a result, we successfully collected data from 55 patients with meticulously preprocessed PPG and ABP data. The primary objective of this effort was to curate a diverse and authentic dataset encompassing various PPG waveform shapes. We randomly extracted 37,500 samples (300 s) from each patient for analysis. Due to the extensive size of the overall database, acquiring the initial data and selecting patients with high-quality waveforms for machine learning posed significant challenges and required substantial storage space. To conserve storage space, we randomly selected 100 patients.

**Figure 3:**
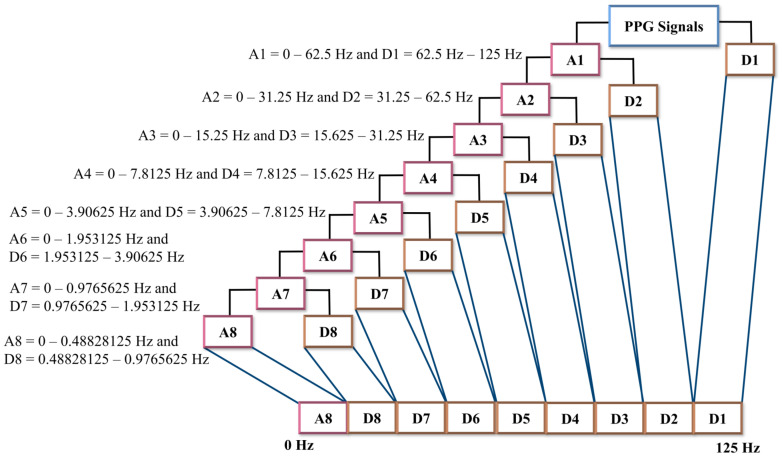
One-dimensional wavelet decomposition. The PPG signals are passed into the LPF to produce an approximation component and are passed into HPF to produce the detail component. In one-dimensional wavelet decomposition, eight-level decomposition generated nine sub-bands, which consisted of one approximation component and eight detail component sub-bands.

Several previous studies used a discrete wavelet decomposition (DWT) filter to minimize PPG signal artifacts and baseline drift [19,20,21]. A one-dimensional (1D) DWT decomposition divides a signal into two frequency components: the low-frequency (LPF) component and the high-frequency (HPF) component. The LPF component is used to extract the approximate coefficient (A), while the HPF component is used to extract the detail coefficient (D) [21,22]. We applied 1D discrete wavelet decomposition (DWT) with Daubechies order four (db4) to mitigate baseline drift and artifacts in the PPG signals. We employed an eight-level decomposition, resulting in nine sub-bands, including one approximation (A) coefficient signal and eight detail (D) coefficient signals. The eight-level DWT composition of the PPG signals with frequency sampling 125 Hz provided the bandwidth sub-band that was sufficient to see the components of the PPG signals. Figure 3 illustrates the frequency range calculation for each level of DWT composition.

The systolic peak in the PPG waveform reflects the maximum blood volume during cardiac circulation, indicating the peak pressure exerted on the arterial wall [5,6]. By considering the interval between consecutive systolic peaks, peak-to-peak measurement captures the pulsatile changes in blood pressure [5]. Thus, the systolic peak-to-peak approach in PPG signals represents a comprehensive component of the systolic and diastolic components of the arterial pulse. The systolic peak-to-peak approach in the PPG signals represents a comprehensive component of the systolic and diastolic components of the arterial pulse. Furthermore, the proposed study by Athaya et al. used overlapping segmentation for each window and achieved superior performance [23]. Therefore, in this study, the PPG signals were segmented into two cycles of systolic peak-to-peak, with one cycle overlapping.

However, the number of samples in the PPG signal over two cycles may vary among individuals, depending on their heart-rate conditions. To address this issue, a spline interpolation method was utilized to achieve uniform sample length in the PPG signal for all data. The most extended sample length of data was 200 samples. Consequently, the samples failing to reach a quantity of 200 were extended through the utilization of spline interpolation. Spline interpolation aims to add new data points between the existing samples. The spline interpolation function yields a series of interpolated values corresponding to the given query points [24].

**Figure 4:**
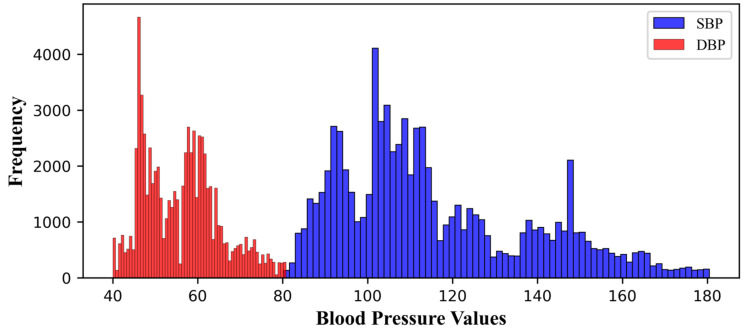
Blood-pressure values distribution.

We removed abnormal BP values (SBP ≥ 200, DBP ≥ 120, SBP ≤ 80, and DBP ≤ 40), based on the blood-pressure range proposed by Chobanian et al. [23,25]. Figure 4 shows the final distribution of systolic and diastolic blood pressure. The minimum and maximum values of SBP were 80.21 and 180.47, respectively, while the minimum and maximum values of DBP were 40 and 80.78, respectively. As a result of preprocessing, we obtained 75,226 signal segments from 55 patients, divided into training sets (60,180), validation sets (12,030), and testing sets (15,045).

Variations in the magnitude and variability of the PPG signal in each patient are caused by the patient’s movements, the patient’s health conditions, or the user device. To assure consistency in data processing, z-score standardization was implemented during the training and assessment phases by following Equation (1).

(1) $$X_{standardization}=\frac{X-mean\;\left(X\right)}{Standard\;Deviation\;\left(X\right)}$$

The average of two PPG pulses can be calculated by adding up all the values in each PPG pulse and dividing by the total number of values, where *X* is the PPG signal. In addition, Equation (2) is used to calculate the standard deviation of two PPG pulses.

(2) $$\sigma =\sqrt{[\frac{\left(\sum {\left(x-\mu \right)}^{2}\right)}{N}}]$$

where *σ* is the standard deviation of the PPG signal, *x* is the PPG signal, *μ* is the average value of the PPG signal, and *N* is the total number in the PPG signal. These calculations reflect how far each value in the PPG pulse is from the average, relatively, in a deviation.

### 2.3. Hyperparameters Tuning for the Proposed Model

Hyperparameter adjustment is a challenging task in deep learning, as it involves selecting the appropriate hyperparameters to optimize the algorithm. Hyperparameter adjustment aims to identify the hyperparameter values that yield models with the best performance that are applicable to various input data problems [26]. However, hyperparameter selection is time-consuming, requiring repeated experiments and evaluations. One commonly used approach is the grid-search method to facilitate the hyperparameter-selection process. Grid search works by systematically testing different combinations of pre-defined hyperparameter values. This method evaluates each combination using cross-validation schemes to determine the most optimal set of hyperparameters. The advantage of grid search resides in its ability to comprehensively explore the hyperparameter space, yielding high learning accuracy and facilitating parallel processing during each machine-learning training session. Nevertheless, it is essential to acknowledge that the exponential growth in hyperparameter combinations necessitates substantial computational resources. The characteristics of the data can significantly influence the appropriate selection of hyperparameters in machine learning. Several studies have investigated the impact of hyperparameters on the performance of machine-learning models [17,22,27,28,29]. Hence, in this research, we constrained the hyperparameter space we explored. We evaluated different optimizers, including Adam, Adadelta, RMSProp, and SGD, and varying batch sizes (32, 64, and 128). Additionally, we considered multiple learning rates (0.001, 0.01, 0.005, and 0.05). The chosen optimizers have demonstrated popularity and effectiveness in machine learning. Including different batch sizes allowed us to understand their influence on model performance. Meanwhile, various learning rates were selected to explore different levels of parameter adjustment in the learning model. 

#### Optimizer, Learning Rate, and Batch Size

Choosing the optimizer is crucial in optimizing performance and predicting results. Selecting the appropriate optimizer can enhance the speed of convergence and improve the accuracy of prediction. However, the efficacy of the optimizer is dependent on other critical factors, such as the learning rate and sample size. The learning rate interacts with the optimizer to determine the number of samples used in each update. A higher learning rate can lead to more rapid convergence, while a lower learning rate can result in slow convergence [30,31,32]. Similarly, a larger batch size can expedite the training process but may prevent the model from capturing subtle patterns in the PPG data. Therefore, the relationship among the learning rate, the optimizer, and the batch size requires careful consideration to achieve the right balance and attain optimal results in deep learning simulations using PPG data.

Among the most commonly used optimizers in the various prediction and classification cases, SGD, RMSprop, Adadelta, and Adam were selected for study. Therefore, we expanded our analysis using Adam, Adadelta, RMSprop, and SGD optimizers [10,27,28,29,33,34,35,36]. We considered the merits of each optimizer, including the SGD [33], RMSprop [29], Adam [37], and Adadelta [29] formulae. However, we only partially depended on the direct application of these optimizers. Instead, we incorporated them into the training process using the grid-search method, which systematically explored combinations of hyperparameters, including learning rate (0.01, 0.001, 0.05, and 0.005) and batch size (32, 64, 128), to identify the optimal model performance.

- The stochastic gradient descent (SGD) optimizer updates the parameters iteratively by subtracting the gradient multiplied by the learning rate, as described in Equation (3):
  (3) $$W_{new}=W_{old}-\alpha \nabla L\;\left(W_{old},\;x_{i},y_{i}\right)$$
  where $W_{new}$ is the update weight, $W_{old}$ the previous weight value, α is the learning rate, and $\nabla L\;\left(W_{old},\;x_{i},y_{i}\right)$ is the gradient loss of function of $L\;\left(W_{old},\;x_{i},y_{i}\right),$ which indicates the direction and magnitude of change required to optimize the model. The process described by this formula is repeated in the proposed deep learning simulation until the model reaches convergence or a state where the loss function is minimized and the prediction of the PPG dataset becomes optimal.

b. Root mean square propagation (RMSprop): The RMSprop optimizer adapts the learning rate for each parameter based on the gradient changes in the previous iterations. RMSprop utilizes the average squared estimation of the previous gradients to adjust the learning rate at each parameter update step. The formula for RMSprop is described in Equations (4) and (5), where ρ represents the forgetting factor (set to 0.9) and t denotes the current time step:
  (4) $$W_{new}=W_{old}-\frac{\alpha }{\sqrt{MeanSqure\left(W,t\right)}}\nabla L\left(W_{old}\right)$$
  (5) $$Mean\;square\;\left(W,t\right)\rho MeanSquare\left(W,\;t-1\right)+\left(1-\rho \right){\left(\nabla L\left(W\right)\right)}^{2}$$

c. Adaptive moment estimation (Adam): Adam is the most commonly used optimization algorithm in deep learning for training models. The Adam optimizer combines momentum optimization concepts and RMSprop to effectively update model parameters during the training process. The Adam optimizer is widely employed in training deep learning models on time series datasets because it accelerates convergence and achieves superior results. The formula for the Adam optimizer is shown in Equation (6):
  (6) $$m_{t}=\rho_{1}m_{t-1}+\left(1-\rho_{1}\right)g_{t}$$
  (7) $$u_{t}=\rho_{1}u_{t-1}+\left(1-\rho_{2}\right)g_{t}{}^{2}$$
  $\mathrm{where}\;\rho_{1}$ and $\rho_{2}$ are the exponential decay rates, g is the gradient. By utilizing Formulae (6) and (7), the correct biases for the first and second moments can be calculated using Equations (8) and (9), respectively:
  (8) $$\hat{m_{t}}=\frac{m_{t}}{1-\rho_{1}t\;}$$
  (9) $$\;\hat{u_{t}}=\frac{u_{t}}{1-\rho_{2}t\;}\;$$

Finally, the parameters of the machine-learning model are updated according to the following Equation (10), where $\varepsilon =10^{-8}$ is the small constant that is used to ensure numerical stability:

(10) $$W_{new}=W_{old}+\Delta W$$

(11) $$\Delta W=-\alpha \frac{\hat{m_{t}}}{\sqrt{\hat{u_{t}}}+\varepsilon }$$

d. Adadelta is an extension from AdaGrad, which is calculated by using Equation (12), where RMS is root mean square error:
  (12) $$W_{i+1}=W_{t}-\frac{RMS\;{\left[\Delta W\right]}_{i-1}}{RMS{\left[g\right]}_{t}}g_{t}\;$$

### 2.4. Proposed Deep Learning Model for Estimating BP 

#### 2.4.1. Long Short Term-Memory (LSTM) Architecture

**Figure 5:**
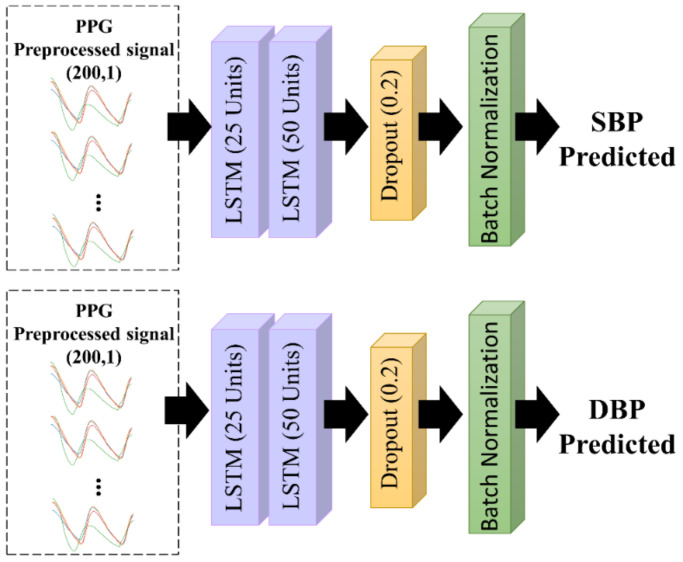
The proposed LSTM architecture.

Recurrent networks (RNNs) are commonly used to analyze time-series data, owing to their ability to store memory accurately and to recognize patterns. However, RNNs cannot predict data stored in long-term memory. Therefore, LSTM is a modification of the RNN, which complements the weakness of the RNN. LSTM can predict information based on past information stored for a long time. Thus, LSTM can remember a collection of information that has been stored for an extended period and delete information that is no longer relevant [38]. The proposed LSTM architecture is shown in Figure 5. We used two LSTM layers, with 25 and 50 units in the first and second layers, respectively. Each unit included an input gate and forget gate, which determined how the information would be added to the cell state. Moreover, an output gate was used to determine the status of the hidden layer. The final LSTM output was the current cell state and the hidden layer state. To avoid overfitting, a dropout rate function of 0.2 and batch normalization were used after the processing of the LSTM units.

#### 2.4.2. LSTM-Based Autoencoder

**Figure 6:**
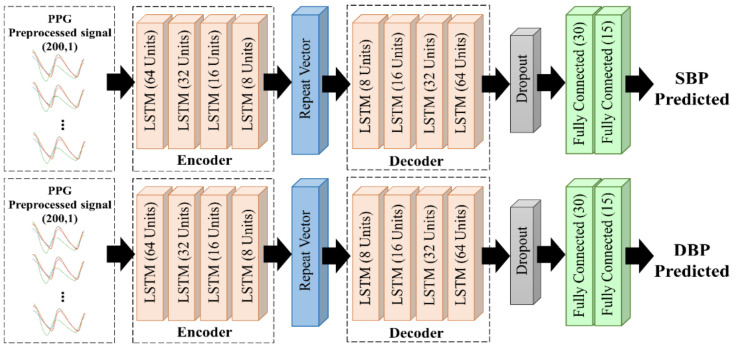
The proposed LSTM–autoencoder architecture.

There are two stages in the autoencoder concept: encoding and decoding, as shown in Figure 6. First, the encoder receives the compressed input data and implements them in the hidden layer. Then, the compressed input data from the previous stage are reconstructed using the decoder stage. As the last layer of the encoder stage does not return a sequence, a repeat vector is required to convert the output into a sequence with the same time-step as the model input. We proposed an LSTM–autoencoder model comprising four LSTM layers each in the encoder and decoder layers. We used a dropout rate of 0.2 and Glorot Normal as the initializer kernel, to avoid overfitting [39].

#### 2.4.3. CNN–LSTM Architecture

**Figure 7:**
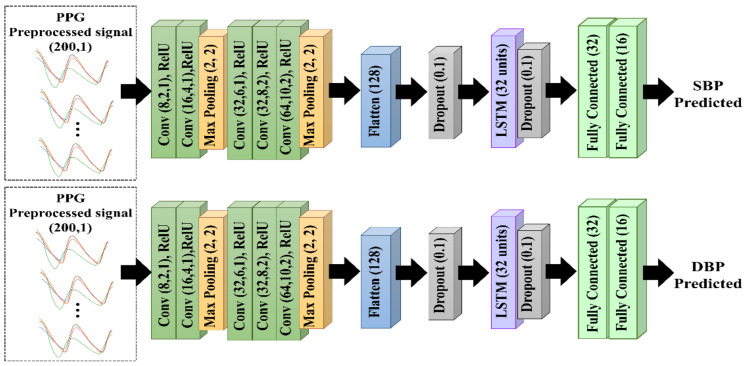
The proposed 1D CNN–LSTM architecture.

As shown in Figure 7, the proposed CNN–LSTM model consisted of five CNN layers for morphological feature extraction and one LSTM layer for temporal feature extraction. They were connected to a dropout layer with a dropout rate of 0.1 to prevent overfitting. The CNN layers consisted of five convolutional layers followed by a max-pooling layer, and each layer used a rectified linear unit (ReLU) as the activation function. Maximum pooling followed the convolutional layer to reduce feature map dimensions and accelerate computation. Furthermore, the feature maps were reshaped by flattening to generate a feature vector. The LSTM layer was then connected to two fully connected layers to predict the SBP and DBP. The CNN method involved feature extraction in the convolution layer and was capable of automatically extracting features. An advantage of the LSTM was its ability to remember long-term sequences because of the increased number of memory cells in the LSTM architecture. Therefore, we combined the CNN and LSTM methods to achieve the optimum performance.

### 2.5. Metrics and Evaluation

In this study, we used mean square error (MSE) as a metric evaluation. Therefore, the proposed model was evaluated with three standard evaluations: those of the Association for the Advancement of Medical Instrumentation (the AAMI), the British Hypertension Society (the BHS), and the Institute of Electrical and Electronics Engineers (the IEEE).

**The IEEE standard:** For analyzing the performance, the IEEE standard was proposed using the MAEs as the parameters, as used in the current standard [40]. As shown in Table 1, an A grade was attained when the mean absolute difference (MAD) ≤ 5 mmHg. The MAE was the average difference between the actual and predicted values, as shown in Equation (13). Here, n is for the data size, $p_{i}$ is the test measurement and $y_{i}$ is the average of reference measurement.

(13) $$MAD=\left(\sum_{i=1}^{n}\left|p_{i}-y_{i}\right|\right)/n\;$$

**British Hypertension Society (BHS) standard:** The BHS is a standard used to assess BP measurement devices and methods. According to the BHS standard, the performance is determined by the absolute error, which is divided into three categories: A, B, and C. If the evaluation score was less than grade C, the study failed to meet the minimum requirements of the BHS standard. As per the standard, the absolute percentage error of prediction must be ≤5, 10, and 15 mmHg to achieve grades A, B, and C, respectively [41].

**Association for the Advancement of Medical Instrumentation (AAMI) standard:** The AAMI standard is used to evaluate SBP and DBP measurement devices and algorithms. This evaluation assesses the mean error (ME) and standard deviation (SD) [42]. As shown in Table 1, the ME should be ≤5 mmHg, and the SD should be ≤8 mmHg. The ME represents the average error between the predicted and actual values, as shown in Equation (14). The estimated values $\hat{y}$ *=* [*y*1, *y*2, …, *y*n] and $y_{i}$ = [*y*1, *y*2, …, *y*n] are the ground truth values and *N* is the total sample size.

(14) $$\mathrm{Mean}\;\mathrm{error}=\frac{1}{N}*\sum_{i=1}^{n}\left(y_{i}-\hat{y}\right)$$

The SD represents the average value used to determine data distribution in a sample. Additionally, it indicates how closely the data is related to the mean. The SD value was used as an indicator of error dispersion [43]. The lower the SD value, the closer the data is to the average value. Conversely, the higher the SD value, the more comprehensive the range of data variations. The SD equation is shown in Equation (15), where *n* is the number of data points in the dataset, *y* = [*y*1, *y*2, *y*3, …, *y*n] is the value of the *i*th point in the dataset, and $\bar{y}$ is the mean value of the dataset.

(15) $$SD=\sqrt{\frac{1}{n-1}\sum_{i=1}^{n}{\left(y_{i}-\bar{y}\right)}^{2}}$$

## 3. Results

This study evaluated the performance of LSTM, LSTM–autoencoder, and CNN–LSTM in estimating SBP and DBP. We used a grid-search method with five-fold cross-validation to acquire the optimal model during the training process. The training data consisted of 65,000 and 65,235 PPG signal segments for predicting the SBP and DBP, respectively. The model was then evaluated using test data consisting of 14,763 and 15,423 PPG segments to predict SBP and DBP, respectively. Table 2 summarizes the prediction performance of each model, based on MAE and SD. 

After implementing the grid-search method, the best hyperparameter for the LSTM model was identified as the AdaDelta optimizer, with an LR of 0.001 and a batch size of 32 for predicting the SBP. Meanwhile, RMSprop was selected as the optimizer for predicting the DBP with an LR of 0.001 and a batch size of 64. Subsequently, for the LSTM-autoencoder, the Adadelta optimizer with an LR of 0.001 and a batch size of 64 was selected as the best parameter for predicting the SBP. Furthermore, the Nadam optimizer with an LR of 0.001 and a batch size of 32 was selected as the best hyperparameter setting for the predicted DBP. For the CNN–LSTM prediction model, the grid-search method selected the Adadelta optimizer with an LR of 0.001 and a batch size of 64 for predicting SBP and the RMSprop optimizer with an LR of 0.001 and a batch size of 64 for predicting DBP. 

**Figure 8:**
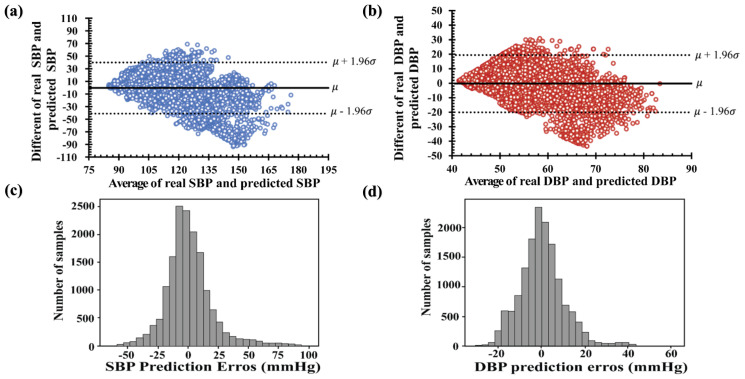
Bland–Altman plots of the proposed LSTM model: (**a**) systolic blood pressure and (**b**) diastolic blood pressure. Error histogram predicted systolic blood pressure (**c**) and diastolic blood pressure (**d**).

The Bland–Altman plots in Figure 8a,b show the average and different values of the real and estimated values of SBP and DBP. The *x*-axis represents pressure ranges of 75 to 180 and 40 to 90 mmHg for SBP and DBP, respectively. The *y*-axis denotes errors of −90 to +70 mmHg for SBP and −40 to +30 mmHg for DBP. The average errors between the real and predicted values were approximately (−50, 100) mmHg for SBP and (−30, 30) mmHg for DBP, as shown in Figure 8c,d. Among the histograms in Figure 8c,d, the deviation of the predicted SBP value was two times higher than the predicted DBP. Therefore, the results of the SBP and DBP predicted using the LSTM failed to satisfy the IEEE, AAMI, and BHS standards, as shown in Table 3. Furthermore, the cumulative percentages of the predicted SBP and DBP falling within the error range of ±5 mmHg were 30.92% and 45.14%, respectively.

**Figure 9:**
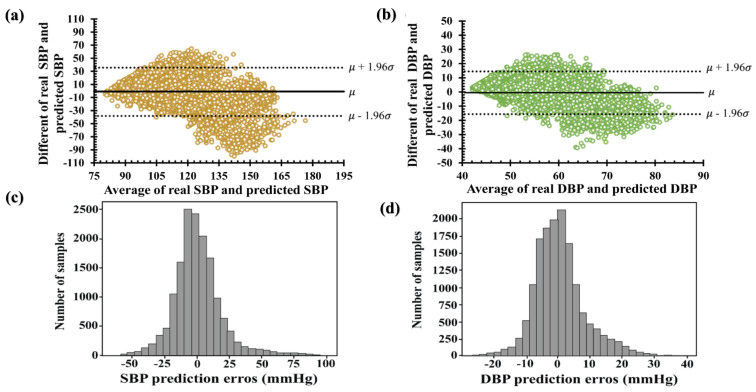
Bland–Altman plots of the proposed LSTM–autoencoder model (**a**) systolic blood pressure and (**b**) diastolic blood pressure. Error histogram predicted systolic blood pressure (**c**) and diastolic blood pressure (**d**).

Figure 9 shows the Bland–Altman plots and histogram error distributions of the real and predicted blood pressures with the LSTM–autoencoder. The Bland–Altman plots in Figure 9a,b reveal that the ME for SBP is −0.94, with a confidence interval of (−38.2 mmHg, 36.3 mmHg), and the ME for DBP is −0.56 mmHg with a confidence interval of (−15.56 mmHg, 14.48 mmHg), respectively. Several SBP values were predicted with high differences, as shown in Figure 9c,d. Therefore, the DBP-predicted performance obtained a B grade in the AAMI evaluation standard and passed the IEEE evaluation standard. Furthermore, in accordance with an error range of ±5 mmHg, the conference cumulative percentages of the estimated SBP and DBP were 26.94 and 56.674, respectively. Based on the BHS protocols, the predicted SBP performance obtained a D grade, and the DBP values obtained a C grade.

**Figure 10:**
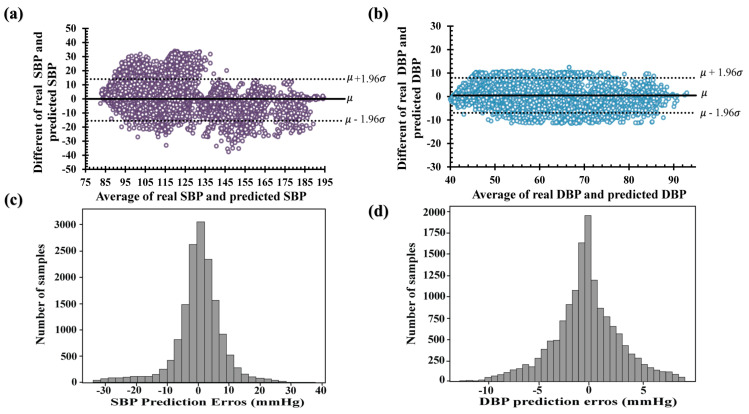
Bland–Altman plots of the proposed CNN–LSTM model (**a**) systolic blood pressure and (**b**) diastolic blood pressure. Error histogram predicted systolic blood pressure (**c**) and diastolic blood pressure (**d**).

Finally, compared to the other LSTM and LSTM–autoencoder models, CNN–LSTM outperformed the other models, as shown in Table 3. The Bland–Altman plots of the predicted SBP and DBP using CNN–LSTM are shown in Figure 10a,b, respectively. The difference between the real and predicted SBPs ranged from −40 to 40 mmHg, while the difference between the real and predicted DBPs ranged from −10 to 10 mmHg. The ME of the SBP difference was −0.13 mmHg, with a confidence interval of (−15.6 mmHg, 15.32 mmHg), and the ME performance for the difference of DBP performance was 0.45 mmHg, with a confidence interval of (−6.8 mmHg, 7.8 mmHg). Therefore, the error distribution was approximately 0 mmHg, as shown in Figure 10c,d. In addition, the deviation in the estimated SBP values was higher than that in the predicted DBP values, as shown in Table 2 and Table 3. The cumulative percentages of the predicted SBP and DBP values were 63.4% and 81.70%, respectively, with an error range of 5 mmHg. According to the BHS evaluation, the predicted values of SBP and DBP obtained grades of B and A, respectively.

## 4. Discussion

The machine-learning approach has been a primary focus in several studies to estimate SBP and DBP using PPG signals. These studies presented innovative and high-performing approaches to optimizing blood-pressure estimation, involving in-depth analysis of various aspects of PPG signals, including using features, raw data, and signal derivatives. The datasets used in these studies differed in terms of the number of patients, preprocessing techniques, prediction algorithms, and evaluation metrics employed. Therefore, we summarized the existing research and compared the performances based on MAE and SD, as shown in Table 2. The obtained performance metrics, including MAE and SD, indicated the level of precision and variation in the BP estimation attained by the proposed method.

Liu et al. used a combination of features extracted from the PPG and second-derivative PPG (SDPPG) domains from the MIMIC II dataset and used support vector regression (SVR) to estimate SBP and DBP. They used 70% of 910 PPG pulse cycles as training data and the remaining 30% as test data. According to the results, the MAEs obtained were 8.54 and 4.34 for estimating SBP and DBP, respectively [44]. Hasanzadeh et al. used 14 PPG features as input into AdaBoost with 200 decision tree estimators and applied 10-fold cross-validation to select the optimal model for measuring SBP and DBP. Based on the BHS evaluation, the performance obtained grades of A and C for predicting DBP and SBP, respectively [8]. Shimazaki et al. used a pulse wave of one beat, velocity plethysmography (VPG), accelerated plethysmography (APG), third-derivative pulse wave, and fourth-derivative pulse wave in a proposed one-dimensional (1D) CNN model to estimate BP. A limitation of their study was that they estimated only the SBP value with SD performances of 13.20, and not DBP [45]. Zhang et al. used a dataset from the University of Queensland Vital Signs Dataset for estimating BP using PPG signals. More than 7000 PPG heartbeats with different blood-pressure values were processed to extract the nine characteristic parameters based on each the maximum and minimum of each pulse wave. Furthermore, they used 75% of the data as training and 25% of the data as testing, and used the SVM model for predicting SBP and DBP, respectively [7]. In addition, Slapničar et al. used short segments of raw PPG, PPG, and PPG” from MIMIC III as temporal-domain inputs into the ResNet architecture. Their study obtained MAE performances of 9.43 and 6.88 for estimating SBP and DBP, respectively. However, their study required a high computational time during the training with ResNet because the full network had to be trained several times [10]. 

The utilization of peak-to-peak PPG (photoplethysmography) signals in machine learning for blood-pressure estimation offers three distinct advantages. First, it provides valuable insights into the systolic and diastolic characteristics of the arterial pulse by measuring the distance between the maximum and minimum peaks within the PPG waveform. Second, peak-to-peak PPG signals exhibit reduced sensitivity to motion artifacts compared to other signal features, as the measurement of peak-to-peak distances primarily focuses on the overall amplitude of the waveform, thereby minimizing the impact of motion artifacts during the estimation process. Finally, peak-to-peak PPG signals can capture the systolic and diastolic components of the arterial pulse, thus offering a comprehensive representation of the blood-pressure waveform [5,6]. Therefore, we used two cycles of the PPG signal peaks as inputs for the proposed model. We developed a non-invasive method for continuous blood-pressure estimation based on the PPG signal from the MIMIC III system using LSTM, LSTM–autoencoder, and CNN–LSTM models with a grid-search method to find the optimal hyperparameters of the proposed model. As presented in Table 2, the proposed CNN–LSTM is superior to the LSTM and LSTM–autoencoder models, because each convolution layer extracts essential features from the PPG signal and improves the quality of the information. 

Using a grid-search approach, this study proposed a machine-learning algorithm with the best hyperparameter settings. Our findings contribute to advancing non-invasive and continuous BP monitoring techniques and, offering potential improvements in patient care and cardiovascular health management. The application of grid search can reduce the processing time compared with the manual consideration of the appropriate parameters from each model prediction and increase the performances even though only PPG signals are used. However, this study has several limitations. First, based on the assessment guideline, we used 55 subjects, which passed only the IEEE standard, which has a minimum of 45 required subjects. However, the AAMI and BHS guidelines require more than 85 participants. Second, more prominent data points are necessary, because the long-term monitoring model of the LSTM performance relies on time-measurement data [46]. The grid-search technique, particularly in CNN–LSTM, offers significant advantage. This method enables comprehensive exploration of the predefined hyperparameter space by systematically and thoroughly searching through all possible combinations. Grid search facilitates the determination of optimal values for each hyperparameter, leading to improved performance. Moreover, tuning the hyperparameter automatically reduces the computational time required for hyperparameter tuning. Therefore, the integration of grid search in CNN–LSTM represents an effective and optimal choice to achieve better performance and efficiency in hyperparameter tuning for deep learning models.

## 5. Conclusions

The development of continuous and non-invasive blood-pressure-measurement methods is a highly intriguing research topic. In this study, we proposed a continuous and non-invasive estimation method for systolic blood pressure (SBP) and diastolic blood pressure (DBP) based on convolutional neural network-long short-term memory (CNN–LSTM), using peak-to-peak signals from photoplethysmography (PPG) to detect early-stage blood-pressure health and to assist in early-stage identification of blood-pressure-related health issues. We also compared the CNN–LSTM method with the classical LSTM and LSTM–autoencoder approaches. Additionally, using optimal hyperparameters obtained through grid-search methodology improved the prediction performance of SBP and DBP. The SBP and DBP estimation results obtained from the CNN–LSTM method complied with the standards set by the Association for the Advancement of Medical Instrumentation (the AAMI), the British Hypertension Society (the BHS), and the Institute of Electrical and Electronics Engineers (the IEEE) when compared to the classical LSTM and LSTM–autoencoder methods. Nevertheless, this research requires validation using a more substantial dataset to verify the clinical feasibility of our proposed model.

## Figures and Tables

**Table 1:** Performance requirements based on evaluations of three standards: the IEEE standard, the BHS standard, and the AAMI standard. MAE: mean absolute difference, CP: cumulative percentage, ME: mean error, SD: standard deviation.

**BHS**	**Grade**	**CP** 5 mmHg	**CP** 10 mmHg	**CP** 15 mmHg	**IEEE**	**Grade**	**MAD (mmHg)**	**AAMI**	**Grade**	**ME (mmHg)**	**SD (mmHg)**
	A	60%	85%	95%		A	≤5		Pass	≤5	≤8
	B	50%	75%	90%		B	5–6				
	C	40%	65%	85%		C	6–7				
	D	Lower than C				D	Lower than C				

**Table 2:** Evaluation of the performances of the proposed method with the prior studies in estimating systolic blood pressure (SBP) and diastolic blood pressure (DBP) using mean absolute error (MAE) and standard evaluation (SD).

Author	Method	Input	Dataset	SBP		DBP	
				MAE	SD	MAE	SD
Proposed work	LSTM	PPG	MIMIC III	14.2	20.7	7.53	10.01
Proposed work	LSTM– Autoencoder	PPG	MIMIC III	13.45	19.01	5.71	7.67
Proposed work	CNN + LSTM	PPG	MIMIC III	3.64	7.04	2.39	3.79
[44]	SVR	PPG	MIMIC II	8.54	-	4.34	-
[7]	SVM	PPG	Queensland	11.6	8.2	7.6	6.7
[10]	Spectro-temporal ResNet	PPG	MIMIC III	9.43	-	6.88	-
[13]	ANN	PPG	MIMIC II	9.74	12.40	4.65	6.29
[12]	RNN	PPG	MIMIC III	12.08	15.67	5.56	7.32
[11]	U-Net	PPG	MIMIC III	5.73	-	3.45	-
[45]	CNN	PPG	Private dataset	-	14.03	-	-
[8]	AdaBoost	PPG	MIMIC II	8.22	10.38	4.17	4.22
[16]	CNN–BiLSTM	PPG	UCI (MIMIC II)	7.85	8.41	4.42	4.80

**Table 3:** Performance evaluation of the proposed model for the estimation of systolic blood pressure (SBP) and diastolic blood pressure (DBP) by using three evaluation standards: the IEEE standard, the BHS standard, and the AAMI standard. MAE: mean absolute error, MAPD: mean absolute percentage differences, CP: cumulative percentages, ME: mean differences, SD: standard deviations.

Assessment Evaluation	IEEE Standard			AAMI Standard		BHS Standards			
	MAD (≤4 mmHg)	MAPD (%)	Grade	ME (<5 mmHg)	SD (<8 mmHg)	CP_5_ (>60%)	CP_10_ (>85%)	CP_15_ (>95%)	Grade
LSTM proposed model									
SBP	14.281	0.12	D	−0.49	20.7	30.92	53.07	67.37	D
DBP	7.53	0.133	C	−0.21	10.01	45.14	72.02	86	C
LSTM–autoencoder proposed model									
SBP	26.94	0.11	D	−0.93	19.01	27	52.5	68.70	D
DBP	5.71	0.01	B	−0.56	7.67	56.67	83.8	92.98	B
CNN–LSTM proposed model									
SBP	5.34	0.04	B	0.13	7.04	63.4	85.9	92.78	B
DBP	2.89	0.05	A	0.48	3.79	81.70	98.28	100	A

## Data Availability

The dataset used in this study can be accessed on the PhysioNet website at https://physionet.org/content/mimic3wdb/1.0/ (accessed on 1 April 2022).
